# Clavicle fixation to reduce short-term analgesia and improve respiratory function in patients with chest wall injuries

**DOI:** 10.1007/s00402-023-04952-5

**Published:** 2023-07-04

**Authors:** Helena Franco, Frances Williamson, Silvia Manzanero, Michael Schuetz

**Affiliations:** 1 Trauma Service, Royal Brisbane and Women’s Hospital, Butterfield Street, Herston, Brisbane, QLD 4029 Australia; 2 Royal Brisbane and Women’s Hospital, Jamieson Trauma Institute, Metro North Health, Butterfield Street, Herston, Brisbane, QLD 4029 Australia; 3 Faculty of Health Sciences and Medicine, Bond University, Gold Coast, Australia; 4 Faculty of Medicine, The University of Queensland, Brisbane, QLD Australia; 5 School of Clinical Sciences, Queensland University of Technology, Brisbane, QLD Australia; 6 Australian Institute of Bioengineering and Nanotechnology, The University of Queensland, Brisbane, QLD Australia; 7 Orthopaedic Department, Royal Brisbane and Women’s Hospital, Butterfield Street, Herston, Brisbane, QLD 4029 Australia; 8 Emergency and Trauma Center, Royal Brisbane and Women’s Hospital, Butterfield Street, Herston, Brisbane, QLD 4029 Australia

**Keywords:** Clavicle fracture, Rib fracture, Clavicle open reduction and internal fixation, Chest wall trauma

## Abstract

**Introduction:**

The objective of this study was to determine if operative fixation of clavicle fractures in patients with non-operatively treated ipsilateral rib fractures is associated with a lower overall analgesic requirement and improved respiratory function.

**Materials and methods:**

A retrospective matched cohort study was conducted involving patients admitted to a single tertiary trauma centre having sustained a clavicle fracture with ipsilateral rib fracture/s between January 2014 and June 2020. Patients were excluded if brain, abdominal, pelvic, or lower limb trauma was identified. 31 patients with operative clavicle fixation (study group) were matched 1:1 to 31 patients with non-operative management of the clavicle fracture (control group) based on age, sex, number of rib fractures and injury severity score. The primary outcome was the number of analgesic types used, and the secondary outcome was respiratory function.

**Results:**

The study group required a mean of 3.50 types of analgesia prior to surgery which decreased to 1.57 post-surgery. The control group required 2.92 types of analgesia, reducing to 1.65 after the date of surgery in the study group. A General Linear Mixed Model indicated that the intervention (operative vs. non-operative management) had statistically significant effects on the number of required analgesic types (*p* < 0.001, $\eta_{{\text{p}}}^{2}$ = 0.365), oxygen saturation (*p* = 0.001, $\eta_{{\text{p}}}^{2}$ = 0.341, 95% CI 0.153–0.529) and temporal decline in daily supplemental oxygen requirement (*p* < 0.001, $\eta_{{\text{p}}}^{2}$ = 0.626, 95% CI 0.455–0.756).

**Conclusion:**

This study supported the hypothesis that operative clavicle fixation reduces short-term in-patient analgesia use and improves respiratory parameters in patients with ipsilateral rib fractures.

**Level of evidence:**

Level III therapeutic study.

## Introduction

Clavicle fractures are common in chest trauma patients, resulting from direct trauma and forces transmitted through the shoulder girdle [1]. It has been demonstrated that additional thoracic injuries are identified in 77% of patients with clavicle fractures, with rib fractures as the most frequent associated injury [2]. A systematic review found both clavicle/s and rib fracture/s occur in 18.6% of patients with blunt chest wall trauma [3].

Clavicle fractures may be treated non-operatively or operatively, most often by open reduction and internal fixation (ORIF). Based on an emerging body of evidence, there has been a trend towards increasing operative management of clavicle fractures. A randomised clinical trial by the Canadian Orthopaedic Trauma Society suggested that operative fixation should be considered for significant displacement, defined as > 100%, shortening of > 20 mm, severe comminution, symptomatic malunion or non-union, floating shoulder and multi-trauma patients [4]. Though this study considered multi-trauma patients, there were no specific recommendations for the operative management in patients with concomitant rib fractures. Two previous small studies have assessed simultaneous operative management of both clavicle and rib fractures in chest wall trauma patients [5, 6]. Langenbach et al. reported that all patients had uncomplicated radiographic union by 12 months [5]. Solberg et al. compared nine patients undergoing both clavicle and rib ORIF to seven patients managed non-operatively, two of whom required delayed operative management due to non-union [6]. A retrospective review comparing operative and non-operative management of floating shoulder and flail chest reported that patients who underwent operative management had a shorter ICU length of stay and time requiring invasive ventilation [7]. To date, no published studies have explored the outcomes of operative management against non-operative management of clavicle fractures with concomitant rib fractures.

Clavicle fractures often result in substantial analgesia utilisation. Weinberg et al. reported that patients who underwent a clavicle ORIF had an overall decrease in opioid use post-operatively compared to those with non-operatively managed fractures [8]. Trauma patients with multiple chest wall injuries often have worse analgesic control than those with isolated injuries [9], and combined clavicle and chest injuries require a sustained increase in analgesia up to 16 weeks post injury [8]. Each additional rib fracture is associated with increased morbidity and mortality due to respiratory complications [10]. It has been hypothesised that a concomitant clavicle fracture may exacerbate the respiratory complications associated with rib fractures [3].

Further evidence is needed to guide the management of patients with a clavicle fracture and ipsilateral rib fracture/s. The objective of this study was to compare overall analgesic requirement and respiratory function between patients who underwent operative fixation of clavicle fractures with non-operatively treated ipsilateral rib fracture/s and patients who underwent non-operative management of both clavicle and rib fractures.

## Materials and methods

### Study design

This is a single-centre, retrospective matched cohort study. The approval was obtained by the institution’s Human Research Ethics Committee prior to data collection (HREC/2020/QRBW/72070).

### Eligibility criteria

The institution’s Trauma Service database was used to identify patients admitted between 1st January 2014 and 30th June 2020. Inclusion criteria were skeletally mature patients aged ≥ 16 years with a unilateral or bilateral clavicle fracture (proximal, middle or distal) and one or more ipsilateral rib fractures, no medical contraindications to general anaesthetic and clinical records for a minimum of 3 months post-injury. Exclusion criteria were pathological clavicle or rib fractures, fractures identified ≥ 28 days after injury and significant other injuries, including severe traumatic brain injury with Glasgow Coma Scale (GCS) score less than 9 or intubated on arrival to the Emergency Department, Abbreviated Injury Scale (AIS) ≥ 2 in pelvic and/or lower limb trauma and significant abdominal injuries requiring laparotomy, traumatic deaths or clinical records for less than 3 months post-injury.

### Matching process

Traditionally, standard management of clavicle fractures at the study site has been primarily non-operative. However, recent practice change has evolved to operative intervention when concomitant rib fractures are present. Due to the change in surgical management, data were available for 31 patients who received operative intervention for the clavicle fracture and met the selection criteria. These patients formed the study group. Patients were matched with the control group on a 1:1 ratio based on sex, age, number of rib fractures, and injury severity score. This matching process was chosen to minimise potential bias due to other factors that may have influenced the surgeon’s treatment choice, for example, patient demographics or injury severity. This matching design additionally led to equal distribution of covariates between the groups. The matching process identified 31 patients from 3657 in the non-operatively managed patient group. These patients formed the matched control group.

### Data collection

A structured chart review of eligible patients was performed to collect relevant data (HF). Sourced data included: characteristics, co-morbidities, analgesic use, respiratory parameters, length of stay and complications. Standard analgesia management at the study site includes the routine use of five categories of in-hospital analgesia: oral analgesia, intravenous opioids, patient-controlled analgesia, regional blocks, and ketamine infusions. In-hospital analgesic medications were recorded as, firstly, the number of analgesic types required each day as discrete data and, secondly, the type of analgesia required each day during hospital admission as categorical data. Respiratory function was measured as continuous data for average daily oxygen saturation (SpO_2_, as a percentage), average daily respiratory rate (breaths per minute) and daily oxygen supplementation (litres per minute). The daily values were calculated as an average for each day. Patient individual identifiers were removed prior to analysis.

### Statistical analysis

Propensity scores were estimated using binary logistic regression analysis. The regression coefficients and odds ratios were interpreted to determine if the study group and matched control group were homogenous with respect to patient characteristics so that the coefficients were close to 0.00 and odds ratios were close to 1.00. Statistical analysis was conducted using the Generalised Linear Mixed Model (GLMM) in IBM SPSS version 24. The random effects in the GLMM were factors that could not be used again if the study was repeated, including the patients and the time spent in the hospital. The fixed effects were research design features that could be used again if the study was repeated, specifically the allocation of patients to groups. Variables investigated included: date of surgery, defined as the number of days between injury and surgery; number of days after the injury; and the intervention (study group vs. matched control group). Partial eta squared ($\eta_{{\text{p}}}^{2}$) ± 95% confidence intervals (CI) were reported to indicate effect sizes, reflecting the relative proportions of the variance in outcome explained by each factor. The total variance explained was indicated by the coefficient of determination (*R*^2^) ± 95% CI.

## Results

Thirty-one patients who underwent operative management of the clavicle fracture were matched with 31 patients (from 3657) who had been managed non-operatively. No patients were excluded during the statistical analysis. Table 1 presents the binary logistic regression results to estimate the propensity scores reflecting equivalent proportions of patients in the study and matched control group for age, sex, number of rib fractures and severity of injury. One patient in each cohort had bilateral clavicle fractures.

**Table 1:** Data used for matching patients

	Study group	Control group	*b*	Standard error (SE)	Wald test statistic	*p*	Odds ratio (OR)	95% CI
Age	47.3 (16–78)	47.6 (20–83)	− 0.005	0.019	0.074	0.786	0.99	[0.96, 1.03]
Sex (male)	83.8%	83.8%	− 0.100	0.729	0.019	0.891	0.91	[0.22, 3.77]
Number of rib fractures	4.7 (1–10)	4.5 (1–9)	0.064	0.131	0.239	0.625	1.08	[0.82, 1.38]
Injury severity score	21.5 (5–45)	21.4 (5–41)	0.002	0.026	0.003	0.953	1.00	[0.95, 1.05]

Age, number of rib fractures and injury severity score are presented as mean (range). Sex is presented as n (%). Binary logistic regression to predict differences between the characteristics of the study group vs the control group

For the study group, the average length of hospitalisation was 10.4 days (range 3–21 days; standard deviation, 5.0), and nine patients required admission to the intensive care unit (ICU). For the matched control group, the average length of hospitalisation was 9.4 days (range 3–25 days; standard deviation, 5.15), and six patients required admission to ICU. The two groups had similar co-morbidities, as outlined in Table 2. The study group had a mean of 0.96 co-morbidities per patient (range 0–5). The matched control group had a mean of 0.7 co-morbidities per patient (range 0–3).

**Table 2:** Co-morbidities

Co-morbidity	Study group	Control group
Hypertension	6 (19.4)	8 (25.8)
Anxiety and/or depression	5 (16.1)	1 (4.5)
Chronic obstructive pulmonary disease	3 (9.7)	2 (6.5)
Hypercholesterolaemia	3 (9.7)	3 (9.7)
Asthma	2 (6.5)	0 (0)
Gastroesophageal reflux disease	2 (6.5)	1 (3.2)
Alcohol dependence	2 (6.5)	1 (3.2)
Hepatitis C positive	1 (3.2)	0 (0)
Opioid dependence	1 (3.2)	0 (0)
Non-metastatic malignancy	1 (3.2)	0 (0)
Obstructive sleep apnoea	1 (3.2)	0 (0)
Heart valve pathology	1 (3.2)	0 (0)
Type two diabetes mellitus	0 (0)	3 (9.7)
Intravenous drug use	0 (0)	1 (3.2)
Previous ischaemic stroke	0 (0)	1 (3.2)
Atrial fibrillation	0 (0)	1 (3.2)

Data is presented as *n* (%)

The study group patients underwent surgery between days 1 and 14 post-injury with a mean of day 5, a median of day 4, and 22 out of 31 patients (70.1%) underwent surgery on or before day 5 post-injury.

## Pain

Two indicators were used to retrospectively assess the degree of pain in both groups of patients. Firstly, the number of analgesic types required each day, which was the primary outcome of this study, was based on the assumption that a need for more types of analgesia would be associated with higher pain severity. Secondly, the type of analgesia used each day during hospital admission. In the study group, the mean number of analgesic types [± 95% confidence intervals (CI)] declined from 3.50 [3.41, 3.59] across all days prior to surgery to 1.57 [1.41, 1.73] across all days from surgery to discharge. Over the same period, in the control group, the mean number of analgesic types declined from 2.93 [2.52, 3.33] across all days before the date surgery was performed in the study group to 1.65 [1.51, 1.79] across all days from the date on which surgery was performed in the study group to discharge. The analysis indicates that the date of surgery had the strongest effect on the temporal changes in the number of required analgesic types (*p* < 0.001, $\eta_{{\text{p}}}^{2}$ = 0.772, 95% CI 0.649–0.856). The second strongest effect was the time (days) after the injury (*p* < 0.001, $\eta_{{\text{p}}}^{2}$ = 0.577, 95% CI 0.396–0.720). The intervention (study group vs. matched control group) had a smaller but still significant effect on the number of analgesic types (*p* < 0.001, $\eta_{{\text{p}}}^{2}$ = 0.365). However, a weak interaction was found between the effect of the intervention and the date of surgery (*p* = 0.025, $\eta_{{\text{p}}}^{2}$ = 0.166, 95% CI 0.031–0.356). The GLMM explained a vast proportion (*R*^2^ = 95.0%; 81.8–97.0%) of the variance in the temporal decline in the number of analgesic types required suggesting potential clinical relevance.

**Fig. 1:**
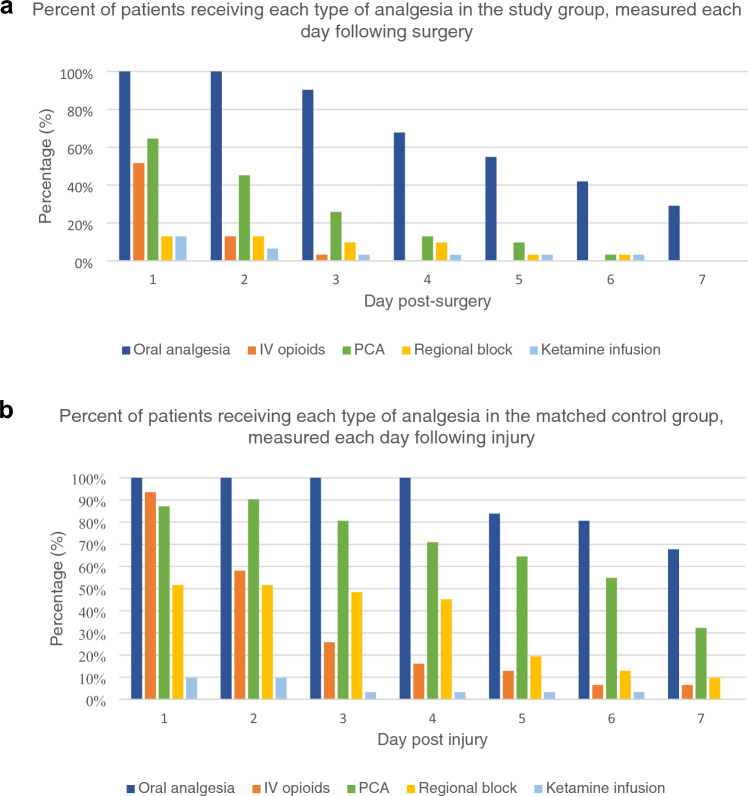
**a** Percentage of patients receiving each type of analgesia in the study group measured each day following surgery. *PCA* patient-controlled analgesia. **b** Percentage of patients receiving each type of analgesia in the matched control group measured each day after injury. *PCA* patient-controlled analgesia

In this study, the most common type of analgesia was oral opioids, followed by patient-controlled analgesia (PCA) and IV opioids. With respect to the type of analgesia used each day during hospital admission, the study group had a reduction in each form of analgesia each day following surgery (Fig. 1a). All study group patients required oral analgesia on the first day after surgery, and this proportion decreased to 29% on day 7 post-surgery. Of the study group patients, 51.6% required intravenous (IV) opioids on day 1 after surgery, and no patients required IV opioids from day 4 post-surgery onwards. PCA was required for 64.5% of patients on day 1 but was removed from all patients by day 7 post-surgery. Regional blocks and ketamine infusions were the least used types of analgesia after surgery, administered to 12.9% of patients on day 1 and all removed by day 7. Overall, all types of post-operative analgesia rapidly deescalated, indicating improvements in pain levels.

Figure 1b demonstrates the in-hospital analgesia used for matched control group patients each day following injury. All patients required oral analgesia for 4 days after the injury, which decreased to 67.7% by day 7. Intravenous opioids were initially required in 93.5% of control patients on day 1 following injury, which rapidly declined to 6.5% of patients by day 6. Within the control group, PCA was commenced in 87.1% of patients following their injury, with one patient commencing PCA on day 2. Only 32.3% of control patients required PCA by day 7 post injury. Regional blocks, administered to 51.6% of control patients following the injury, declined to 9.7% of patients by day 7. Finally, ketamine infusion was administered to 9.7% of control patients from day 1 following injury, with no patients requiring a ketamine infusion by day 7.

### Respiratory function

Respiratory rate was compared between the two groups. In the study group, the mean respiratory rate changed from 14.3 (14.2, 14.3) breaths per minute averaged over the pre-surgery period to 15.3 (15.4, 15.7) after surgery. In the control group, the mean respiratory rate (± 95% confidence intervals) changed from 15.1 (15.0, 15.2) breaths per minute, averaged over the period prior to the date of surgery in the study group, to 15.3 (15.2, 15.4) after that date. The number of days from injury had little or no effect on the temporal changes in respiratory rate (*p* = 0.279, $\eta_{{\text{p}}}^{2}$ = 0.042, 95% CI 0.000–0.187). The intervention (study group vs matched control group) also had little effect (*p* = 0.125, $\eta_{{\text{p}}}^{2}$ = 0.082, 95% CI 0.002–0.250), as did the date of surgery (*p* = 0.096, $\eta_{{\text{p}}}^{2}$ = 0.086, 95% CI 0.002–0.256). A weak interaction was identified between the effect of the intervention and the date of surgery (*p* = 0.038, $\eta_{{\text{p}}}^{2}$ = 0.144). The GLMM explains a moderate proportion (*R*^2^ = 46.9%; 27.5–63.7%) of the variance in changes in the daily respiratory rate. Besides, as the average respiratory rates between groups lie within normal parameters (12 to 16 breaths per min), these findings may not be clinically significant.

In the study group, the mean daily oxygen saturation (± 95% confidence interval) increased from 96.1% (95.9, 96.3) averaged over the pre-surgical period to 96.9% (96.3, 97.4) averaged from the date of surgery to discharge. In the control group, the mean daily oxygen saturation increased from 96.4% (96.1, 96.7) prior to the average day when surgery was performed in the study group to 97.1% (96.8, 97.3) in the remaining days to discharge. The GLMM analysis indicated that the time (days) after the injury had the strongest effect on the temporal changes in the daily oxygen saturation (*p* < 0.001, $\eta_{{\text{p}}}^{2}$ = 0.756, 95% CI 0.627–0.846). The intervention (study group vs. matched control group) had a smaller but significant effect (p = 0.001, $\eta_{{\text{p}}}^{2}$ = 0.341, 95% CI 0.153–0.529) similar in size to the effect of the date of surgery (p = 0.001, $\eta_{{\text{p}}}^{2}$ = 0.347, 95% CI 0.158–0.535). However, a weak interaction was found between the effect of the intervention and the date of surgery (*p* = 0.025, $\eta_{{\text{p}}}^{2}$ = 0.154). The GLMM explained a large proportion (*R*^2^ = 79.2%; 67.8–86.9%) of the variance in the improvement in oxygen saturation.

In the study group, the mean daily supplemental oxygen requirement (± 95% confidence interval) decreased from 2.4 (2.2, 2.5) L/min pre-surgery to 0.6 (0.03, 1.2) after surgery. In the control group, the mean daily supplemental oxygen requirement declined from 1.8 (1.9, 2.3) L/min in the period prior to the average date surgery was performed in the study group to 0.3 (0.1, 0.6) L/min after that date. The GLMM statistics indicated that the time (days) after the injury had a strong effect on the temporal changes in supplemental oxygen requirement (*p* < 0.001, $\eta_{{\text{p}}}^{2}$ = 0.574, 95% CI 0.392–0.718). The intervention (study group vs. matched control group) also had a strong effect (*p* < 0.001, $\eta_{{\text{p}}}^{2}$ = 0.626, 95% CI 0.455–0.756), but the effect of the date of surgery was weaker ($\eta_{{\text{p}}}^{2}$ = 0.346, *p* < 0.001). No significant interaction was found between the effect of the intervention and the date of surgery (*p* = 0.347, $\eta_{{\text{p}}}^{2}$ = 0.032). The GLMM explained a vast proportion (*R*^2^ = 92.2%; 87.3–95.2%) of the variance in the decline in daily supplemental oxygen requirement.

### Complications

Two out of 31 patients in the study group required a re-operation for subsequent removal of the implant (6.5%). No cases of metalware failure, superficial or deep space infection or symptomatic non-union occurred in the study group. One patient required intravenous antibiotics for right lower lobe pneumonia. In the matched control group, one patient required a delayed open reduction and internal fixation procedure 12 months following injury for symptomatic non-union (3.1%). One patient developed skin irritation under the sling.

## Discussion

This study describes the association between the number of administered analgesic types and the management, either operative or non-operative, of clavicle fractures in patients with concomitant rib fractures (*p* < 0.001, $\eta_{{\text{p}}}^{2}$ = 0.365). The results support the study hypothesis and previous literature demonstrating a decrease in analgesia use in patients with clavicle fixation compared to those managed non-operatively [8].

The reduction in analgesia use between the study and control group is hypothesised to be related to the role that clavicle stability plays in chest wall function. The presence of progressive clavicle fracture displacement in the setting of ipsilateral rib fracture/s may result in sustained pain syndromes [3]. The effects of group and time on the categories of analgesic types required could not be estimated with the GLMM because the data were not directly comparable, as the timeline for the various types of analgesia in the matched control group relates to days post-injury, whereas the timeline for the types of analgesia in the study group is days post-surgery, as necessitated by the varying times to surgery in the group of patients undergoing operative treatment. It is interesting to note that the study group recorded a higher initial number of types of analgesia recorded of 3.50 [3.41, 3.59] compared to the control group of 2.93 [2.52, 3.33] before the date of surgery. While outside the scope of this study, previous literature has demonstrated factors associated with increased use of analgesia, such as Williamson et al., reporting higher morphine milliequivalent use in patients with rib fracture displacement over seven days [11]. A prospective study could be considered to elucidate factors associated with increased pain and whether pain may be considered a relative indication for operative fixation in this patient cohort.

Respiratory function, as measured by respiratory rate, oxygen saturation and daily supplemental oxygen requirements, was compared between the study and matched control group. The results revealed statistically significant differences between the two groups with respect to the temporal improvement in oxygen saturation (*p* = 0.001, $\eta_{{\text{p}}}^{2}$ = 0.341, 95% CI 0.153–0.529) as well as the temporal decline in the daily supplemental oxygen requirement (*p* < 0.001, $\eta_{{\text{p}}}^{2}$ = 0.626, 95% CI 0.455–0.756). There was also a small effect on daily respiratory rate (*p* = 0.125, $\eta_{{\text{p}}}^{2}$ = 0.082, 95% CI 0.002–0.250). Given the robustness of the statistical models in explaining the observed variance, the evidence suggests that operative treatment of clavicle fractures in patients with concomitant rib fractures may be clinically relevant. Previous studies have suggested that clavicle and rib fractures are associated with pain-related respiratory complications [3]. Solberg et al. reported that three out of seven patients who underwent non-operative management for both clavicle and rib fracture/s developed pneumonia, and two patients developed bacteraemia [6]. While supporting literature is lacking, the authors hypothesised that the clavicle serves as a splint for the upper chest wall and that clavicle fixation may assist in stabilising the chest wall to improve respiratory function. As compromised respiratory function may be one factor influencing the clinical decision to proceed to operative intervention, the results of this study support the notion that operative intervention may improve overall respiratory function compared to non-operative management.

The benefits of lower in-hospital analgesic requirements must be balanced against the risk of complications associated with clavicle ORIF. In this study, the re-operation rate for the removal of the implant was 6.5%. This is lower than a previous retrospective study which reported a re-operation rate of 24.6%, most commonly for the removal of metalwork [12]. In the matched cohort, one patient required delayed ORIF for symptomatic non-union (3.1%).

In this retrospective matched cohort study, the propensity scores using binary logistic regression analysis indicated that the study group and matched control cohorts were sufficiently homogenous, and therefore, no sampling bias was revealed. The authors note that the small cohort size limits the results of this study, though this was dictated by the small number of patients undergoing operative management who met the eligibility criteria within the 6-year study period. Furthermore, potential confounding factors, such as the number of ribs fractured, displacement of the clavicle fracture or presence of a pneumothorax, were not accounted for. Co-morbidities, such as pre-existing respiratory disease may influence the daily oxygen saturation and oxygen supplementation required. As patients with respiratory disease were not excluded, these may influence the findings. Another source of bias may be that the operative or non-operative treatment may have been influenced by the surgeon’s preference or other patient factors not considered in the matching process such as severity of pain, noting that patients were not matched according to their analgesic requirements as at the date surgery was performed in the operative cohort. This may have influenced the higher mean number of analgesic types in the surgery, compared to the control group prior to surgery. Consideration should be given to conducting future research utilising this study to develop the adequate power calculation for the design of a prospective randomised controlled trial to ascertain whether operative treatment improves patient post-injury recuperation compared to non-operative treatment. A prospective study would allow for enhanced assessment of pain using a numeric pain rating scale.

The study supported the hypothesis that operative fixation is associated with decreased number of in-patient analgesic types required and reduces daily supplemental oxygen requirements in patients with chest wall injuries.
